# Discovery of MAO-B Inhibitor with Machine Learning, Topomer CoMFA, Molecular Docking and Multi-Spectroscopy Approaches

**DOI:** 10.3390/biom12101470

**Published:** 2022-10-13

**Authors:** Linfeng Zheng, Xiangyang Qin, Jiao Wang, Mengying Zhang, Quanlin An, Jinzhi Xu, Xiaosheng Qu, Xin Cao, Bing Niu

**Affiliations:** 1 School of Life Science, Shanghai University, Shanghai 200444, China; 2 Shanghai General Hospital, Shanghai Jiao Tong University School of Medicine, Shanghai 200080, China; 3 Department of Chemistry, School of Pharmacy, Air Force Medical University, Xi’an 710032, China; 4 Institute of Clinical Science, Zhongshan Hospital, Shanghai Medical College, Fudan University, Shanghai 200444, China; 5 National Engineering Laboratory of Southwest Endangered Medicinal Resources Development, Guangxi Botanical Garden of Medicinal Plants, Nanning 530023, China

**Keywords:** Alzheimer’s disease (AD), monoamine oxidase B (MAO-B) inhibitors, machine learning, molecular docking, fluorescence quenching

## Abstract

Alzheimer’s disease (AD) is the most common type of dementia and is a serious disruption to normal life. Monoamine oxidase-B (MAO-B) is an important target for the treatment of AD. In this study, machine learning approaches were applied to investigate the identification model of MAO-B inhibitors. The results showed that the identification model for MAO-B inhibitors with　K-nearest neighbor(KNN) algorithm had a prediction accuracy of 94.1% and 88.0% for the 10-fold cross-validation test and the independent test set, respectively. Secondly, a quantitative activity prediction model for MAO-B was investigated with the Topomer CoMFA model. Two separate cutting mode approaches were used to predict the activity of MAO-B inhibitors. The results showed that the cut model with q^2^ = 0.612 (cross-validated correlation coefficient) and r^2^ = 0.824 (non-cross-validated correlation coefficient) were determined for the training and test sets, respectively. In addition, molecular docking was employed to analyze the interaction between MAO-B and inhibitors. Finally, based on our proposed prediction model, 1-(4-hydroxyphenyl)-3-(2,4,6-trimethoxyphenyl)propan-1-one (LB) was predicted as a potential MAO-B inhibitor and was validated by a multi-spectroscopic approach including fluorescence spectra and ultraviolet spectrophotometry.

## 1. Introduction

Alzheimer’s disease (AD) [1], the most common cause of senile dementia worldwide, is a degenerative disorder of the central nervous system characterized by memory loss and dysfunctions of language and behavior. Although the pathogenesis of AD is not completely clear, several hypotheses about AD pathogenesis, such as the free-radical injury hypothesis [2], amyloid peptide hypothesis [3], cholinergic hypothesis [4] and Tau hyper-phosphorylation hypothesis [5], are used as the main basis for its treatment. Therefore, there are several potential drug targets such as acetylcholinesterase [6], amyloid-β protein [7] and tau protein [8], which are used for the treatment and amelioration of the disease.

Monoamine oxidases A and B (MAO-A and MAO-B) are flavin adenine dinucleotide (FAD)-dependent enzymes [9] which play important roles in the metabolism of biogenic amines in the central nervous system and in peripheral tissues. MAO-A inhibitors can be used as anti-depressants, whereas MAO-B inhibitors are mainly used in the treatment of neurodegenerative diseases such as AD. MAO-B is an important mitochondrial enzyme [10] which metabolizes β-phenethylamine [11]. Studies suggested that reactive astrocytes in the brain of an AD mice model aberrantly and abundantly produce an inhibitory glio-transmitter, γ-aminobutyric acid (GABA) through MAO-B, and this leads to an abnormal release of GABA through the bestrophin 1 channel [12]. The released GABA reduces the normal flow of information by acting on presynaptic GABA receptors. Therefore, the researchers proposed that selective inhibition of GABA synthesis and release by MAO-B inhibitors may be an effective therapeutic strategy for treating memory impairment in AD patients [13]. Subsequently, several effective MAO-B inhibitors, such as selegiline [14,15] and rasagiline [16], have been shown to be effective in retarding the neurodegeneration normally seen in cases of AD. However, the side effects, including anxiety, hallucinations and dyskinesia, are also obvious [17].

With the circumstances that machine learning has developed rapidly, in silico approaches provide vast help in modern drug discovery. This work describes the discovery of new MAO-B inhibitors with computational approaches. First, machine learning approaches, including SVM, KNN, C4.5, random forest, random tree, AdaBoost and bagging were applied to analyze MAO-B inhibitors qualitatively and predict whether a compound had the ability to inhibit MAO-B. Then, Topomer CoMFA was used to predict the activity of the inhibitors and to identify their stereo-electronic requirements, as well as to reveal the structural factors essential to the interactions with their substrate. Additionally, molecular docking technology was also performed to predict the interactions between MAO-B and MAO-B inhibitors. Finally, 1-(4-hydroxyphenyl)-3-(2,4,6-trimethoxyphenyl)propan-1-one (also called LB) was proposed as a potential MAO-B inhibitor based on the MAO-B inhibitor identification model, Topomer CoMFA model and molecule docking. The in silico findings were further validated with multi-spectroscopy approaches, including fluorescence spectra and UV-vis absorption spectra.

## 2. Materials and Methods

### 2.1. Data Preparation

In this study, a dataset containing 278 inhibitors [18,19,20] from the references (supporting Information SI) and 185 non-inhibitors from the DUD database (http://dud.docking.org, Accessed on 19 January 2019) (supporting Information SII) were collected. A total of 464 compounds containing inhibitors and non-inhibitors were divided into a training set (390 compounds including 251 inhibitors and 139 non-inhibitors) and a test set (73 compounds including 27 inhibitors and 46 non-inhibitors) randomly. Among the 278 inhibitors, 227 inhibitors with activities were selected to build an activity prediction model with Topomer CoMFA. Molecular descriptors were calculated by Sybyl x to characterize compounds. (supporting Information SIII) According to the chemical and physical properties of molecules, these molecular descriptors can be divided into three categories: electric field parameters, structural parameters and thermodynamic parameters. These parameters were used as candidate variables of input variables for feature screening.

### 2.2. Topomer CoMFA

The Topomer CoMFA method, which combines the Topomer technique and CoMFA technology, overcomes the alignment problems associated with CoMFA [21,22]. The partial least squares (PLS) regression method was employed to build the Topomer CoMFA model, and the leave-one-out (LOO) cross-validation was used to evaluate the model. Additional details regarding the Topomer CoMFA can be found in the references [23,24,25].

### 2.3. Molecular Docking

The molecular docking of compounds with MAO-B was simulated by using the Surflex-Dock [26] module in SYBYL-X-2.0. The protein crystal structure of MAO-B (PDB ID: 1GOS) [27] was obtained from the protein data bank (http://www.rcsb.org./pdb, accessed on 16 May 2021). During the protein preparation procedure, water molecules and ligands were removed, and hydrogen atoms were added. In addition, the terminal treatment of the protein resulted in the addition of a charge.

### 2.4. Fluorescence Spectra

LB (2 × 10^−8^ M) was added to MAO-B (1.5 μM)with a micro-injector, 1 μL each time (the cumulative volume was less than 10 μL), and the equilibration time was 5 min. The emission spectrum of MAO-B at 280–450 nm was recorded at 305, 310 and 315 K, respectively. The fluorescence intensity corresponding to the maximum emission wavelength is defined as F.

Three-dimensional fluorescence spectra of the MAO-B and the LB-MAO-B complexes (2:1, molar ratios) were obtained in the excitation wavelength range of 200–480 nm and the emission wavelength range of 220–500 nm.

### 2.5. UV-Vis Absorption Spectra

The ultraviolet absorption spectra of MAO-B and LB before and after the reaction in the range of 200 to 800 nm were recorded. Next, 1μL of LB solution (2 × 10^−4^ M) was added successively to 3 mL of MAO-B solution (6.7 × 10^−8^ M) with a micro-injector, and the solution was allowed to stand for 4 min. In addition, the absorption spectrum of LB (5 × 10^−8^ M) was recorded.

### 2.6. Ultraviolet Spectrophotometry

Sample group: a group where benzylamine was used as a substrate for MAO-B, 190 μL of MAO-B solution and 460 μL LB solution were added to 50 μL potassium phosphate buffer (100 mM) at 37 °C. After incubation for 20 min, 100 μL of 98.1 g·L^−1^ substrate benzylamine was added and shaken at 37 °C for 60 min, after which 60% of perchoric acid was added to stop the reaction. Then, 3 mL of cyclohexane was added, vortexed for 2 min and extracted with benzaldehyde by centrifuging at 10,000× g for 5 min. The value of A was determined at a wavelength of 242 nm.

Blank control group: MAO-B solution + benzylamine, and all the other steps were the same as the sample group.

Complete inhibition group: MAO-B solution + benzylamine immediately after the perchloric acid step, and the subsequent steps were the same as the product group.

Positive control group: MAO-B solution + benzylamine + selegiline, and all the other steps were as for the product group.

The formula used for calculating the inhibition rate is as follows:

Inhibition rate (%) = (A _blank control group_ − A _value of sample group_)/(A _blank control group_ − A _total inhibition group_) × 100% (1)

## 3. Results

### 3.1. Feature Selection and Construction of MAO-B Inhibitor Prediction Model

Molecular descriptors can be used to describe physicochemical and geometric properties of compounds. In this study, 45 molecular descriptors, including electronic, steric and thermodynamic parameters, were calculated by using the ChemOffice software [28], and these molecular descriptors were used to characterize the compounds.

**Figure 1:**
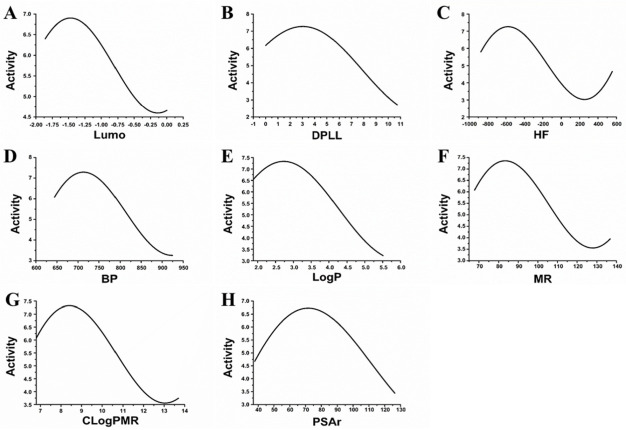
The relationship of activity of different molecule descriptors.

A feature subset containing eight molecular descriptors was obtained based on a correlation-based feature subset (CFS) selection search method combined with a best first search (BFS) algorithm. These eight molecular descriptors were LUMO energy (Lumo), dipole length (DPLL), heat of formation (HF), boiling point (BP), logP, molar refractivity (MR), CLogP molar refractivity (CLogPMR) and polar surface area (PSAr). Sensitivity analysis was applied to these descriptors to evaluate how they affected the activities of MAO-B inhibitors (Figure 1).

Based on the optimal features subset, typical algorithms such as AdaBoost, bagging, K-nearest neighbors (KNN), random forest, random tree, naïve Bayes and C4.5 were applied to build the MAO-B inhibitor prediction model. As a result, prediction accuracies of 85.1–95.9% for the 10-fold cross-validation test were obtained. Among them, the prediction accuracy with KNN outperformed the other machine learning methods. Although the result of the 10-fold cross-validation test was adequate, it was not good enough for evaluating the prediction model as the KNN classifier might over-fit the data. An independent test set was therefore employed to validate the reliability of the classifier. The results showed that the prediction accuracy of the independent test set was 95.2% (Table 1).

### 3.2. Topomer CoMFA Prediction Model

The training set was used to build Topomer CoMFA models by fragmenting compounds into R1 and R2 groups, and the test set was further employed to evaluate the stability and predictive ability of the model. Two Topomer CoMFA models were generated by cutting methods. The Topomer CoMFA Model 2, which had higher q^2^ and r^2^ values, was selected for use in analyzing and predicting the activities of MAO-B inhibitors (Table 2). The experimental and predicted pIC_50_ are listed in Table 3.

**Figure 2:**
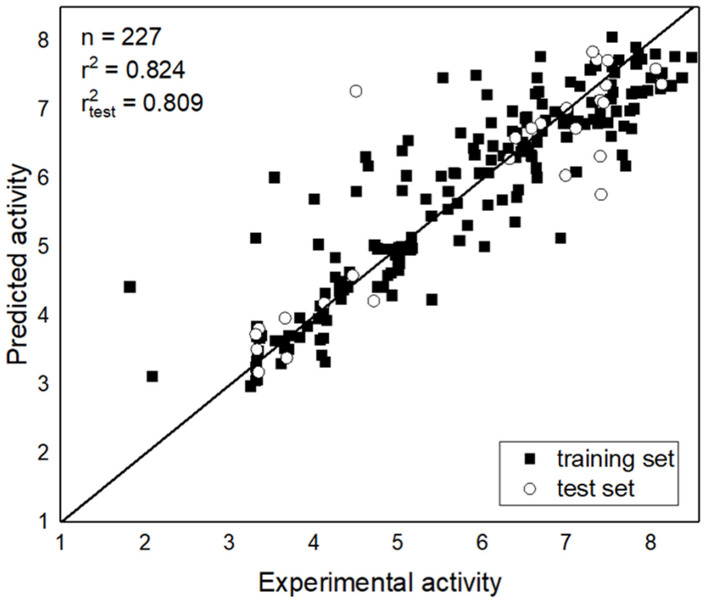
Experimental data versus predicted data from the Topomer CoMFA Model 2.

The plots of experimental data versus predicted activity are shown in Figure 2. The independent test set was applied in order to evaluate the model, and the points were also depicted on the diagonal graph, showing that the model can be used to predict the activities of MAO-B inhibitors. The LOO cross-validated q^2^ value was 0.612. The r^2^ values of the training and test sets were 0.824 and 0.809, respectively.

**Figure 3:**
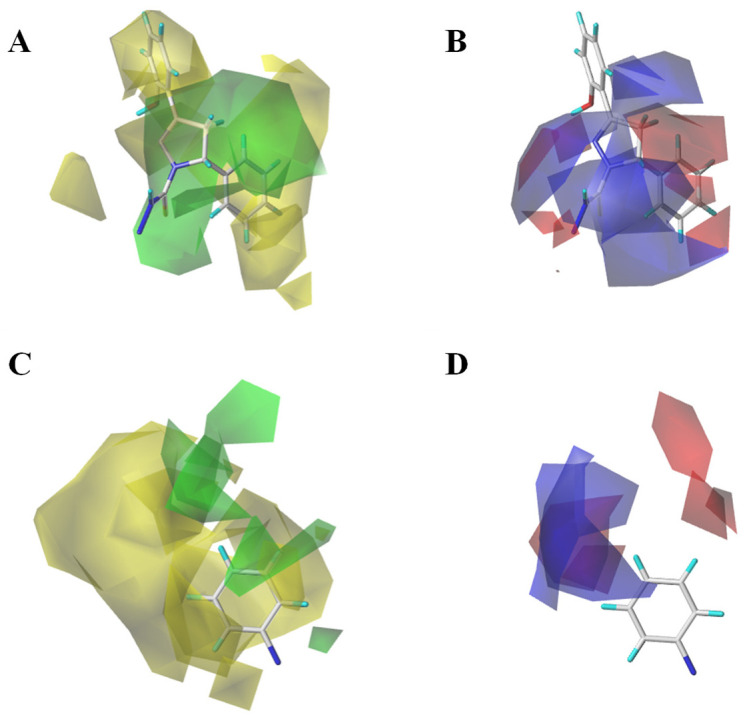
3D contour maps of Topomer CoMFA Model 2 for R1 (**A**,**B**) and R2 (**C**,**D**) of compound 63. ((**A**,**C**) represent steric contour maps. (**B**,**D**) represent electrostatic field maps.).

Additionally, the steric and electrostatic contour maps of the R1 and R2 groups were obtained. Compound 63, with high activity, was selected to study the steric QSAR of MAO-B inhibitors. As Figure 3 shows, the steric field contours are shown in yellow and green, whereas the electrostatic field contours are shown in red and blue. In the steric contour maps, the green and yellow regions indicate that introducing large and small volume groups can improve the compound’s activity, respectively. In the electrostatic field, the red and blue areas show that adding the positive and negative charged groups can improve the compound’s activity, respectively.

### 3.3. Simulation of the Interaction between Compounds and MAO-B

**Figure 4:**
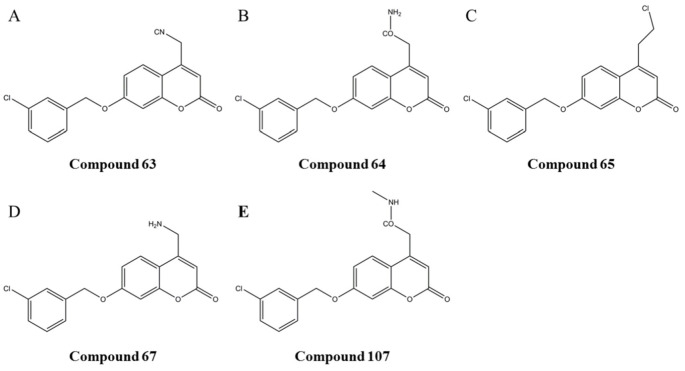
The structures of potential compounds

**Figure 5:**
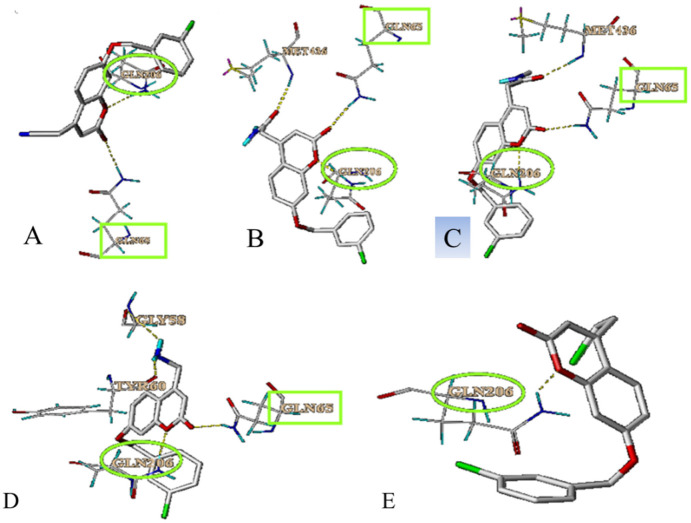
The docking results of inhibitors with MAO-B. (**A**). Compound 63 with MAO-B at GLN206 and GLN65. (**B**). Compound 64 with MAO-B at GLN206 and GLN65. (**C**). Compound 65 with MAO-B at GLN206 and GLN65. (**D**). Compound 67 with MAO-B at GLN206 and GLN65. (**E**). Compound 107 with MAO-B at GLN206. Hydrogen bonding is depicted as yellow dashed lines.

Compounds 63, 64, 65, 67 and 107 (whose molecular structures are shown in Figure 4) were used for molecular docking with MAO-B. As shown in Figure 5, these five compounds can bind to MAO-B through hydrogen bonds. In addition, all of them can interact with GLN206, which was related to the catalytic activity of MAO-B [29]. The compounds **63**, **64**, **65** and **67** can also interact with GLN65 of MAO-B.

### 3.4. Prediction and Validation of the Interaction between Potential Compounds and MAO-B

#### 3.4.1. Predicting and Activity Evaluation of LB

**Figure 6:**
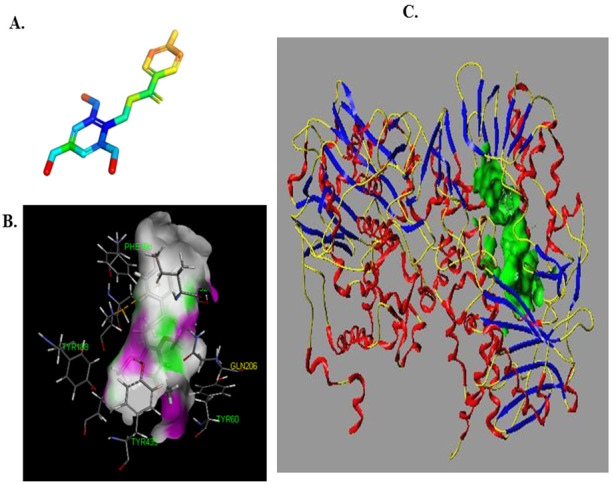
The docking results of LB with MAO-B and the binding pock of MAO-B. ((**A**). The structure of LB, (**B**). The binding pocket of MAO-B, (**C**). The interaction of MAO-B and LB).

Based on the aforementioned MAO-B inhibitor identification model and Topmer CoMFA model, LB (Figure 6A) was predicted to be a potential inhibitor (pIC_5 =_ 4.02).

The interaction between LB with MAO-B was also evaluated with a molecular docking approach. The results thus obtained are shown in Figure 6, where we can see that the binding of LB to MAO-B was mainly through hydrogen bonds, and that there are three binding sites between the LB and MAO-B. Meanwhile, the score of molecular docking was also calculated. The “TotalScore” was higher than 4 (TotalScore = 6.65), which means the interaction between the small molecule and the macromolecule is very strong. Accordingly, this suggests that the interaction between LB and MAO-B is very strong.

Subsequently, the ultraviolet spectrophotometry was used to validate the predicted result. The IC_50_ of selegiline as a positive control to MAO-B was also measured. The IC_50_ of LB and selegiline was 2.448 ± 0.09 and 2.352 ± 0.08 nM, respectively, and the results of the positive control are consistent with other reports.

#### 3.4.2. Fluorescence Quenching of MAO-B

**Figure 7:**
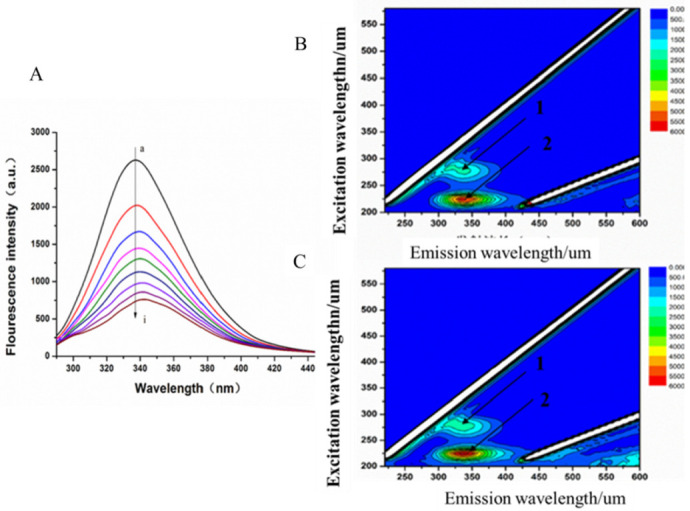
The fluorescence quenching of MAO-B by LB. (**A**). The fluorescence emission spectra of MAO-B-LB with an excitation wavelength of 280 nm, a→i were the fluorescence spectra with 0→8 μL LB added, (**B**). 3D fluorescence spectra of MAO-B, (**C**). 3D fluorescence spectra of MAO-B-LB.).

The fluorescence emission spectra of MAO-B after the action of 3 temperatures (305, 310 and 315 K) with LB and LB alone were recorded, and the emission spectra at 310 K are shown in Figure 7. When the excitation wavelength was set to 280 nm, the fluorescence emission spectra of LB and MAO-B were found. From Figure 7, it can be seen that MAO-B had a strong fluorescence peak when excitation wavelength was 280 nm, while LB had a very weak internal fluorescence under the same experimental conditions (with no LB lines seen). With an increased concentration of LB, the fluorescence intensity of MAO-B gradually decreased, and the decrease became negligible. This shows that there was an interaction between LB and MAO-B, and the combination with LB led to saturation. Under the action of LB, the maximum emission wavelength of MAO-B had a slight red shift (335.8 to 339.2 nm), indicating that LB could induce changes in the microenvironment at 342.4 nm.

According to the fluorescence spectra obtained after adding different concentrations of LB at different temperatures (305 K, 310 K and 315 K), the fluorescence intensity of MAO-B before and after adding LB was recorded as F0 and F, respectively, and F0/F was calculated. The specific data are listed in Table 4.

Fluorescence quenching refers to quenching between the fluorescent and solvent molecules. Fluorescence quenching can be classified as static and dynamic quenching. To confirm the quenching mechanism more closely, we analyzed the fluorescence quenching of MAO-B at different temperatures (305, 310 and 315 K) at an excitation wavelength of 280 nm using the Ster–Volmer equation:

(2) $${\;F}_{0}/F=1+k_{q}{\;\tau }_{0}\left[Q\right]=1=K_{\mathrm{sv}}\left[Q\right]\;$$

where F_0_ and F are the fluorescence intensity before and after the quencher, respectively, kq is the rate constant of the fluorescence quenching process, [Q] is the concentration of the quencher, K_sv_ is the quenching constant and τ_0_ is the life expectancy of a fluorescent molecule (generally 10^−8^ s) in the absence of a quencher [30,31].

**Figure 8:**
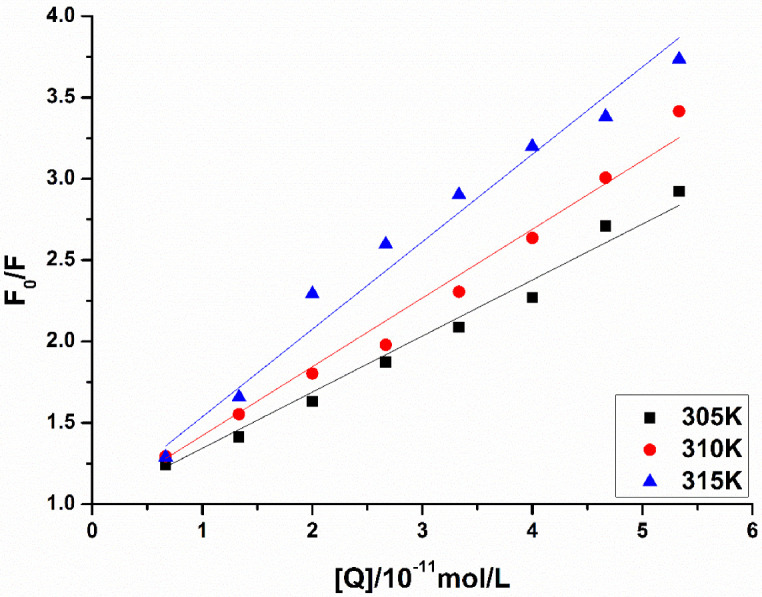
The unmodified Stern–Volmer curves of MAO-B fluorescence quenched by LB.

The Stern–Volmer curves of the fluorescence quenching of MAO-B by LB at different temperatures are displayed in Figure 8, and the quenching constant, K_sv_ and correlation coefficient of MAO-B obtained by the linear equation are shown in Table 5. It was found that the K_sv_ increased as temperature increased. Kq values were 2.79 × 10^10^, 3.38 × 10^10^ and 4.55 × 10^12^ L/( mol·s) at 305, 310 and 315 K, respectively, and these values were almost equal to the maximum quenching rate constants of the biomolecule (2.0 × 10^10^ L/(mol·s)) [32]. All of these results demonstrated that the fluorescence quenching of MAO-B by LB was dynamic quenching, which caused the reaction between the quenching agent and the fluorescent material molecules in the ground state.

### 3.5. Calculation of the Binding Constant and Binding Point

Static fluorescence quenching means that a fluorescent donor molecule and a fluorescent quencher molecule combine to form a ground state complex by means of intermolecular forces. The complex would exhibit a certain structure that is non-fluorescent, which leads to the phenomenon of a decrease in fluorescence. The supposed fluorescence quenching of this type of protein would be static quenching. The binding sites (n) can be determined by the following equation:

(3) $$\mathrm{lg}\left[\left(F_{0}-F\right)/F\right]=\mathrm{lgK}+n\;\mathrm{lg}\left[Q\right]\;$$

The binding affinity of drugs and MAO-B would then be decided by the binding constant. The magnitude of the value reflects the binding strength, and it has a direct impact on the distribution and elimination of the drug from the body. Thus, the binding constant would dictate the intensity and duration of the action of that drug.

**Figure 9:**
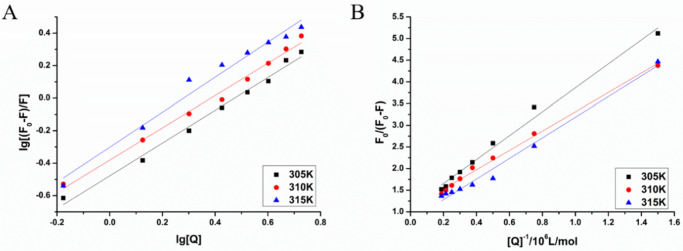
Double logarithmic curves (**A**) and the modified Stern–Volmer curves (**B**) of MAO-B fluorescence quenched by LB.

For n ≈ 1, Equation (4) can be used to calculate the binding constant (K) between the drug and the protein [33]:

(4) $$F_{0}/\left(F_{0}-F\right)=1+K^{-1}{\left[Q\right]}^{-1}$$

where K and n are the binding constant and the number of binding sites, respectively. The corresponding double logarithmic curve (A) and modified Stern–Volmer curves (B) are shown in Figure 9. As shown in Table 6, the number of binding sites of LB is approximately equal to 1. This indicates that MAO-B has a single binding site for LB to interact, and the binding constant will be reduced by a temperature increase that coincides with a change in the quenching constant.

### 3.6. The UV-Vis Absorption Spectra of the LB and MAO-B System

UV-vis absorption spectroscopy [34,35] is a simple and effective way to detect protein conformational changes and complex formation. Thus, the quenching mechanism of the LB and MAO-B was further verified by the UV-vis absorption spectroscopy method.

**Figure 10:**
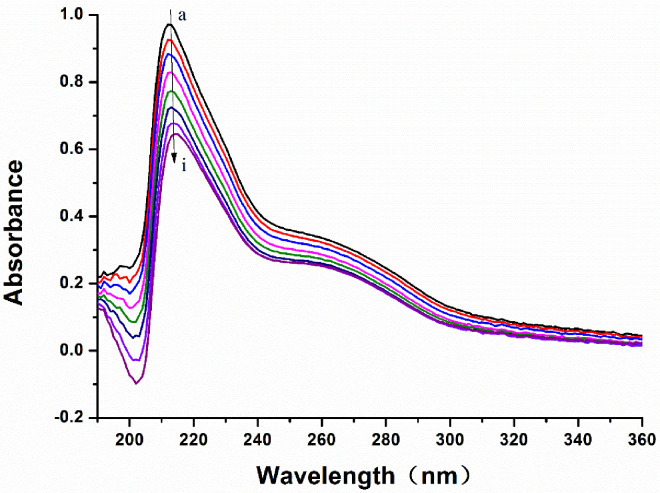
UV–vis absorption spectra of MAO-B in the presence of different LB concentrations. a→i were the UV–vis absorption spectra with 0→8 μL LB added.

From Figure 10, it can be observed that MAO-B has a strong absorption in the wavelength range of 200 to 240 nm, in which the bands observed can reflect the information relating to the protein backbone. The intensity of the absorption peak of MAO-B at 210 nm decreased greatly with the increase in LB concentration, and the absorption peak had a slight red shift. MAO-B has a weaker absorption peak at 280 nm, which is similar to the main absorption peaks of tyrosine and tryptophan residues normally seen with respect to conjugated double bonds. For LB, the absorption peak intensity decreased, but the decrease was minimal at 210 nm. The results showed that the reaction of LB and MAO-B formed a base state complex and caused static quenching. The protein has a slight red shift (from 212 to 214 nm), suggesting that MAO-B could be induced to change the microenvironment of the polypeptide backbone by LB.

## 4. Discussion

It is necessary for a good prediction model to select appropriate features because of the usual existence of irrelevant features. In this case, a subset containing eight features was used to build the MAO-B inhibitor prediction model. The prediction accuracy of the model was deemed to be very good, as it included both a training set and an independent test set. The results showed that the original data contained some redundant features. Thus, feature selection is a necessary step during the building of a useful prediction model.

According to the results of the cross-validation, there was a relationship between the eight descriptors used (Lumo, DPLL, HF, BP, LogP, MR, CLogPMR and PSAr) and the inhibitory activities of MAO-B inhibitors. The sensitivity analysis was further applied to reflect on the relationship between these descriptors and the inhibitor activities directly. For example, Figure 1A shows the relationship between the activity and Lumo. When the Lumo was approximately −0.15 and −1.48, the activity was at the maximum and minimum, respectively. Figure 1B shows the relationship between the activity and DPLL. When the DPLL was approximately 3.07, the activity value was at its peak. Figure 1C shows the relationship between the activity and HF. When the HF was approximately −581.08 and 263.38, the activity values were at the maximum and minimum, respectively. Figure 1D shows the relationship between the activity and BP. When the BP was approximately 712.82, the activity value was at its peak. Figure 1E shows the relationship between the activity and LogP. When the LogP was approximately 2.70, the activity value was at its peak. Figure 1F shows the relationship between the activity and MR. When the MR was approximately 83.27 and 128.32, the activity values were at the maximum and minimum, respectively. Figure 1G shows the relationship between the activity and CLogPMR. When the CLogPMR was approximately 8.35 and 12.99, the activity values were at the maximum and minimum, respectively. Figure 1H shows the relationship between the activity and PSAr. When the PSAr was approximately 72.16, the activity value was at its peak.

The use of a MAO-B inhibitor prediction model indicated that these eight molecular descriptors are able to classify whether a compound is capable of acting as a MAO-B inhibitor. Moreover, these eight molecular descriptors may be related to the activity of the MAO-B.

The activities of MAO-B inhibitors are related to the integrity of the pharmacophore [36]. In the Topomer CoMFA model, the pharmacophore is related to cutting style, which is important for the model’s predictive performance [17,37,38]. In the Topomer CoMFA analysis, the training set is split into two fragments. If the fragmentation is complete, the input structures are standardized and the topomers are generated. All of the topomers share the same identical substructure. If the same identical substructures in the test set are recognized, the model’s predictive ability is promising. The presence of identical substructures is considered as the pharmacophore. In this study, the compounds were fragmented into R1 and R2 groups, with the two models being obtained based on different fragmentation procedures. Model 2, with the highest q^2^ and r^2^ values, was selected as the final model for further subsequent evaluation.

The active site in Model 2 was modified based on the active site in R1 of Model 1, which contributed to the model’s predictive ability (Table 2). Thus, we can speculate that R1 and R2 groups in Model 1 have identical substructures. Generally, a substrate interacts with a receptor through its active pharmacophore. Active pharmacophores have a particular conformation which interacts with key amino acid residues that are the active residues in the active pocket of MAO-B. The molecule is often divided into the active pharmacophore and other structures which are regarded as having the same identical substructures.

In order to further enhance the biological activities of MAO-B inhibitors, the Topomer CoMFA model provides alternative possibilities for modifying MAO-B inhibitors. Compound **63** (Figure 3A) was chosen to study the effects of R1 and R2 groups on the activity. In the R1 group, large and negatively charged groups in the chlorobenzene ring may increase the compound’s biological activity (Figure 8). In the R2 group, small groups with a positive charge on the propiononitrile may also increase the compound’s biological activity (Figure 3). Similar studies have reported that Topomer CoMFA was used in designing xanthine oxido-reductase inhibitors [23]. Additionally, it can also be used in virtual screening for identification of novel antagonists [39].

Molecular docking is used as a method of predicting the interaction sites between the compounds and MAO-B. As shown in Figure 5, there were binding sites between these five compounds and MAO-B. Five compounds can interact with GLN206, which is one of the active sites of MAO-B. Compounds **64**, **65** and **67** also can interact with GLN206. Therefore, we hypothesized that GLN206 might be a new active site of MAO-B.

Reliable prediction model has the capacity to correctly predict potential candidate drugs. In this study, a virtual screening was applied based on our MAO-B inhibitor identification and activity models. As a result, LB was predicted as a potential MAO-B inhibitor. Our experiment also showed that LB inhibits MAO-B well. According to the experimental results of the fluorescence spectrum, compound LB can also cause good fluorescence quenching of MAO-B. Molecular structure and chemical environment are important factors affecting the emission fluorescence and fluorescence intensity of substances. In biological macromolecules, higher numbers of aromatic hydrocarbons or conjugated double bonds lead to strong fluorescence produced by organic compounds. Meanwhile, the nature of substituents also has a great impact on the fluorescence intensity of phosphors. Substituents on a benzene ring will lead to the displacement of maximum absorption wavelength and the change of fluorescence peak. Generally, electronic groups, including—NH2, -Oh, -OCH3, -nhch3 and -n (CH3) 2, can enhance the fluorescence, while electron-absorbing groups, including—Cl, -Br, -I, -nhcoch3, -NO2 and -COOH, will weaken the fluorescence. Compound LB has several -OCH3, and when -OCH3 is connected with the benzene ring, it can be used as an electron donor group only if it forms a para-position effect. In the molecular structure of compound LB, the methoxy group is an electron-absorbing group, so compound LB has stronger fluorescence quenching of MAO-B, which also shows that compound LB has a greater impact on the aromatic amino acids in MAO-B molecule. In addition, as the structure of compound LB is symmetrical, the charge distribution is also uniform and symmetrical, which cause smaller dipole moment. Therefore, the polarity of compound LB is small, while MAO-B focuses on the decomposition and deamination of non-polar aromatic amine phenylethylamine, which may be the reason for the better inhibition effect of L. H-bond force is an important force for the formation of stable protein ground complex, and compound LB has a better inhibitory effect on MAO-B.

The results of molecule docking showed that LB binds to MAO-B at Phe168, CYS 172, ILE198, GLN206 and TYR435 through H-bond. Figure 5 showed that GLN206 is a main amino acid residue which could interact with high activity inhibitors. Hence, GLN206 could be regarded as an important site for inhibitors. Interestingly, our experiments of the fluorescence quenching of MAO-B showed that there is only one binding site for LB to interact with MAO-B, which is different from the docking results. The possible explanation may be that tryptophan (try), tyrosine (Tyr) and phenylalanine (PHE) are the only components of natural amino acids that can emit fluorescence, which can be determined by the fluorescence method. In our molecule docking study, Phe168 was one of the acting sites among the five amino acid residues. Hence, in the fluorescence quenching experiments, the fluorescence intensity decreased, and only one site was concluded.

## 5. Conclusions

In this study, MAO-B inhibitor and Topomer CoMFA prediction models were built to analyze MAO-B inhibitors. Firstly, the MAO-B inhibitor model was built to predict whether a compound was an inhibitor or a non-inhibitor. The accuracy of the MAO-B inhibitor model, using the 10-fold cross-validation and independent set tests, was 94.1% and 88.0%, respectively. Then, a Topomer CoMFA model was built based on MAO-B inhibitors. Two models were obtained by altering different molecular bonds. As a result, Model 2, with higher q^2^ and r^2^ values, was selected for use to predict MAO-B inhibitors. Additionally, a series of similar chemical inhibitors were selected in order to study the interacting sites between MAO-B and MAO-B inhibitors using a molecular docking tool. This resulted in GLN206 and GLN65, which were considered to play crucial roles in the MAO-B activity. Finally, LB was predicted and validated as a potential MAO-B inhibitor. In conclusion, we hope this work will be helpful for the future design of novel drugs against AD.

## Figures and Tables

**Table 1:** The results of the prediction accuracy with eight molecular descriptors using different machine learning methods.

Methods	10-Fold Cross-Validation Test			Independent Set Test		
	SN (%)	SP (%)	ACC (%)	SN (%)	SP (%)	ACC (%)
Naïve Bayes	90.5	67.3	81.7	96.3	53.6	67.5
SVM	95.2	66.0	83.9	100	53.6	68.7
KNN	94.8	93.1	94.1	100	82.1	88.0
C4.5	96.0	87.4	92.7	100	82.1	88.0
Random Forest	96.4	87.4	92.9	96.3	76.8	83.1
Random Tree	92.0	89.9	91.2	96.3	76.8	83.1
AdaBoost	97.2	89.3	94.1	96.3	75.0	81.9
Bagging	98.0	81.8	91.7	96.3	75.0	81.9

**Table 2:** Results from the two topomer CoMFA model studies.

Dataset	Topomer CoMFA Model 1	Topomer CoMFA Model 2
Cutting model	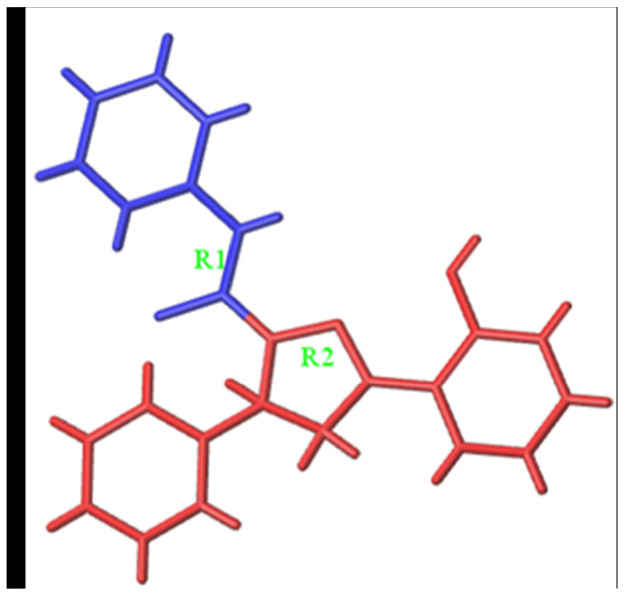	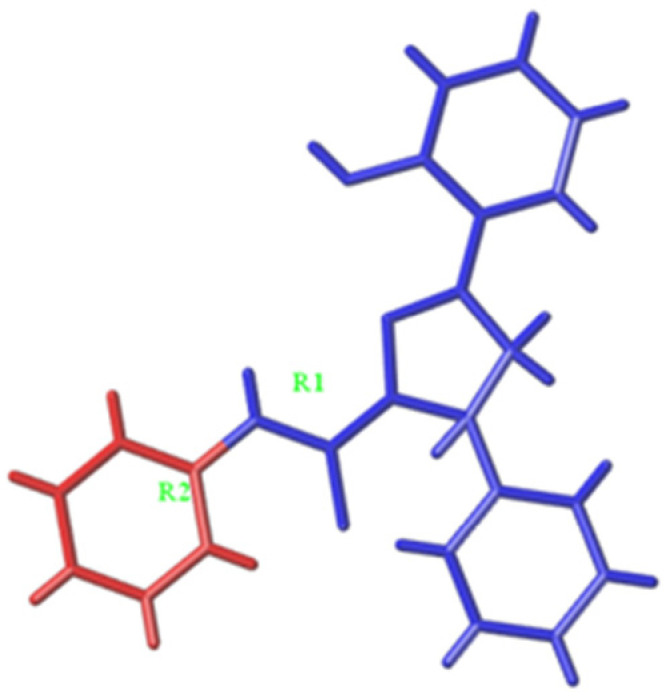
q^2^	0.579	0.612
r^2^	0.752	0.824
r^2^_test set_	0.856	0.809

Note: The different colored parts represents the sub-structure of compounds after cutting.

**Table 3:** Experimental and predicted pIC_50_ for Topomer CoMFA Model 2.

Compound	Exp	Pre	Compound	Exp	Pre
Training set					
1	3.32	3.76	132	6.64	7.48
2	3.33	3.50	133	6.64	6.55
3	3.35	3.69	134	6.62	7.24
5	3.30	3.27	135	6.59	6.33
6	3.37	3.73	136	6.57	6.35
7	3.31	3.15	137	6.54	6.47
10	3.31	3.71	138	6.52	6.91
12	4.11	4.09	139	6.52	6.83
13	4.05	3.97	140	6.49	6.43
15	4.15	3.94	141	6.47	6.54
16	4.11	3.69	142	6.46	6.40
17	4.09	3.44	143	6.42	5.84
19	3.32	3.86	144	6.40	6.68
20	3.31	3.35	145	6.39	6.32
21	3.30	3.06	146	6.35	6.99
23	3.32	3.08	147	6.10	6.82
24	3.32	3.75	148	6.10	6.28
25	3.70	3.73	149	6.07	6.10
27	3.54	3.64	150	6.05	7.23
28	3.65	3.54	151	6.02	5.02
29	3.70	3.52	152	5.96	6.09
31	3.65	3.64	153	5.90	6.35
32	3.61	3.31	154	5.89	6.46
33	4.40	4.43	155	5.72	5.11
35	4.91	4.64	156	5.70	5.65
36	4.30	4.37	157	5.66	6.10
37	4.99	4.80	158	5.59	5.82
39	4.82	4.43	159	5.58	5.56
40	5.82	5.33	160	5.52	7.48
41	7.77	6.74	161	5.50	6.05
43	7.54	7.65	162	5.40	4.25
44	6.06	5.62	163	5.33	5.71
45	7.68	6.78	164	5.17	5.00
47	6.68	7.78	165	5.16	5.00
48	5.04	5.83	166	5.15	5.16
49	5.68	6.09	167	5.14	4.97
51	6.31	6.34	168	5.10	6.05
52	7.32	6.88	169	5.05	6.42
53	7.28	7.60	170	5.04	5.01
55	4.00	5.71	171	5.02	4.91
56	6.38	5.38	172	5.00	5.00
57	6.63	6.16	173	5.00	4.68
59	6.66	7.27	174	5.00	4.83
60	8.28	7.79	175	4.96	4.90
61	7.85	7.83	176	4.92	4.31
63	7.80	7.03	177	4.88	4.98
64	7.52	7.17	178	4.88	4.60
65	7.62	7.74	179	4.82	4.43
67	7.82	7.68	180	4.82	4.43
68	7.89	7.25	181	4.82	4.43
69	7.96	7.30	182	4.79	4.44
71	6.35	6.70	183	4.76	4.43
72	5.95	6.59	184	4.75	4.99
75	7.00	6.86	185	4.72	5.04
77	8.11	7.32	186	4.64	6.19
78	8.24	7.36	187	4.42	4.65
79	6.12	6.48	188	4.35	4.39
81	6.94	6.95	189	4.33	4.51
82	6.90	6.99	190	4.32	4.25
83	6.24	6.34	191	4.30	4.42
85	7.21	6.81	192	4.25	4.57
86	7.13	6.85	193	4.25	4.86
87	4.61	6.32	194	4.13	4.35
89	6.50	6.89	195	4.13	3.34
90	7.34	7.65	196	4.07	4.15
91	5.39	5.47	197	4.07	3.67
93	7.55	7.26	198	4.05	5.05
94	5.11	6.57	199	3.92	3.86
95	5.01	4.77	200	3.82	3.99
97	6.23	5.70	201	3.82	3.70
98	4.50	5.83	202	3.52	6.03
99	7.57	7.55	203	3.30	5.14
101	5.74	6.68	204	3.24	2.99
102	8.48	7.78	205	2.08	3.13
103	8.05	7.82	206	1.81	4.43
105	6.30	6.45	207	8.37	7.48
106	8.13	7.55	208	8.36	7.48
107	7.89	7.75	209	8.36	7.48
109	5.92	7.51	210	8.36	7.48
110	7.82	7.92	211	8.00	7.48
111	7.54	8.08	212	7.82	7.67
113	6.40	5.74	213	7.82	7.27
114	7.37	6.81	214	7.77	7.24
115	7.30	7.12	215	7.74	7.00
116	7.15	7.35	216	7.70	6.19
117	7.11	6.11	217	7.66	6.35
118	7.05	7.42	218	7.62	7.74
119	7.01	6.99	219	7.62	7.74
120	7.01	6.99	220	7.62	7.74
121	7.00	6.99	221	7.59	6.99
122	7.00	6.82	222	7.52	6.63
123	7.00	6.62	223	7.52	7.17
124	6.96	6.81	224	7.52	7.37
125	6.92	5.14	225	7.52	7.17
126	6.85	6.98	226	7.52	7.17
127	6.74	6.86	227	7.48	6.82
128	6.70	6.70	228	7.40	6.96
129	6.70	7.09	229	7.40	7.14
130	6.64	6.03	230	7.40	7.14
131	6.64	6.56	231	7.40	7.14
Test set					
4	3.32	3.52	62	7.11	6.74
9	3.35	3.82	66	7.40	7.14
14	4.12	4.19	70	6.69	6.80
18	3.34	3.19	76	7.44	7.11
22	3.31	3.74	80	6.58	6.74
26	3.66	3.97	84	6.39	6.60
30	3.68	3.40	88	7.00	7.03
34	4.71	4.22	92	8.06	7.60
38	4.46	4.59	96	7.41	5.77
42	8.13	7.38	100	4.50	7.28
46	7.36	7.74	104	7.47	7.37
50	6.32	6.29	108	7.49	7.73
54	7.40	6.33	112	7.31	7.85
58	6.99	6.05			

**Table 4:** The fluorescence intensity at different temperatures of LB before and after the addition of MAO-B.

[Q] (×10^−11^ mol/L)	305 K		310 K		315 K	
	F	F_0_/F	F	F_0_/F	F	F_0_/F
0.00	2836	/	2636	/	2344	/
0.67	2282	1.24	2034	1.30	1819	1.29
1.33	2006	1.41	1697	1.55	1414	1.66
2.00	1739	1.63	1462	1.80	1136	2.29
2.67	1514	1.87	1332	1.98	1003	2.60
3.33	1360	2.09	1143	2.31	897.8	2.90
4.00	1249	2.27	999.8	2.64	814.3	3.20
4.67	1047	2.71	876.6	3.01	770.2	3.38
5.33	970.4	2.92	771.7	3.42	697.4	3.74

**Table 5:** The quenching constant (K_sv_) and correlation coefficient of MAO-B.

T(K)	K_sv_ (×10^2^ L/mol)	K_q_ (×10^10^ L/mol s^−1^)	R
305	2.79	2.79	0.874
310	3.38	3.38	0.902
315	4.55	4.55	0.932

**Table 6:** The binding sites (n) and the binding constant (K) of the inaction of MAO-B and LB.

T(K)	Ka (×10^10^ L/mol)	n	R
305	2.8834	1.0080	0.995
310	2.3267	0.9930	0.995
315	2.1500	1.0767	0.989

R^a^ is the linear correlated coefficient.

## Data Availability

Not applicable.

## Supplementary Materials

The following supporting information can be downloaded at: https://www.mdpi.com/article/10.3390/biom12101470/s1, File S1: Supporting Information SI; File S2: Supporting Information SII; File 3: Supporting Information SIII.
